# Towards nano-mechanical simulations of ceramics containing realistic defects *via* machine-learning potentials: the example of TiB_2_

**DOI:** 10.1039/d6nr00466k

**Published:** 2026-06-10

**Authors:** Chunhui Du, Shuyao Lin, Nikola Koutná, Paul Heinz Mayrhofer

**Affiliations:** a Institute of Material Science and Technology, TU Wien Vienna Austria chunhui.du@tuwien.ac.at; b Department of Physics, Chemistry, and Biology (IFM), Linköping University Linköping Sweden

## Abstract

Transition metal diboride (TMB_2_) ceramics combine high hardness with excellent thermal and chemical stability, making them attractive for protective coating applications. TMB_2_s commonly grow as largely off-stoichiometric—containing vacancies or other simple crystallographic defects—and a particularly essential question is how such defects alter the response to mechanical loads at the nanoscale. We exploit molecular dynamics (MD) equipped with here-trained machine-learning interatomic potential (MLIP) to reveal effects of point and planar defects during nano-mechanical tests of TiB_2_, being a representative of the most common AlB_2_-type TMB_2_s. MLIP training consists of active learning on configurations from finite temperature *ab initio* molecular dynamics, including equilibrium and uniaxially loaded structures as well as various defective and/or extremely strained environments. Transferability to near-indenter-tip environments is achieved *via* on-the-fly training on extrapolative clusters from simple nanoindentation runs. Following MLIP's validation, we simulate room-temperature nanoindentation of TiB_2−*x*_ structures, where B sub-stoichiommetry is realized by disordered B vacancies, single- and double-planar defects previously revealed by electron microscopy. A somewhat non-intuitive prediction is that TiB_2−*x*_ structures can exhibit hardness comparable to the ideal TiB_2_, challenging traditional assumptions about weakening effects of sub-stoichiometry. This behavior is ascribed to Ti-rich planar defects, contrarily to vacancies which, at the same chemistry, notably deteriorate mechanical properties. We hypothesize that similar effects may be expected for other group 5–6 transition metal diborides.

## Abstract 2

This study uses machine-learning interatomic potentials (MLIP) and molecular dynamics to simulate nanoindentation on defective TiB_2_. Results reveal that Ti-rich planar defects may preserve hardness of TiB_2_, unlike boron vacancies.

## Graphical abstract

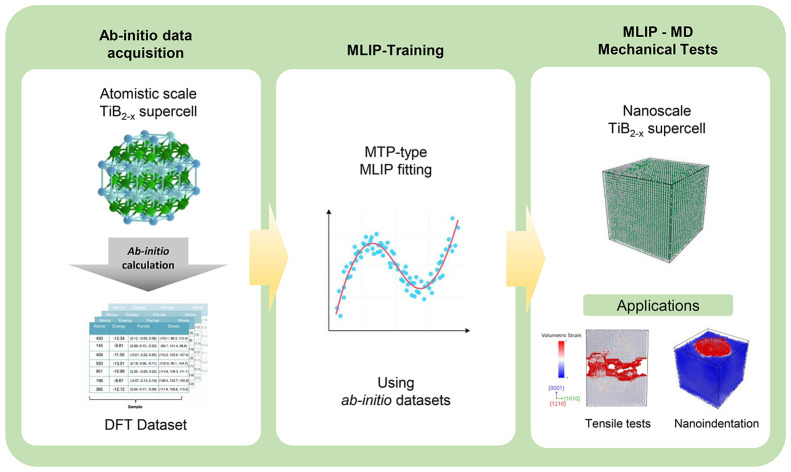

## 1 Introduction

Due to their high melting points, excellent hardness and distinguished chemical stability, transition metal diboride (TMB_2_) thin films have been widely used in the field of cermat and wear-resistant materials.^1–4^ TMB_2_ s typically adopt layered hexagonal structures with the AlB_2_-type (*α*; group IV–V TMs), WB_2_-type (*ω*, group VI TMs), and ReB_2_-type (*γ*, group VII TMs) phase prototype,^5^ where the phase preference may be tailored *via* synthesis conditions—especially for TMB_2_ s exhibiting low energy barriers between phase polymorphs (*e.g.*, NbB_2_ and TaB_2_ ^4,5^)—or *via* shear-induced transformations (*e.g.*, TaB_2_ and WB_2_ ^6^). TiB_2_ has been the most widely researched diboride, with hardness of 30–50 GPa and elastic modulus of 450–600 GPa.^7–11^ This significant spread of reported mechanical properties is seen not only in TiB_2_ but in many other diborides,^12–14^ reflecting various synthesis conditions which promote deviations from the ideal 1 : 2 TM-to-boron ratios.^15,16^ However, experimentally resolving the effects of individual defect types on mechanical response is highly challenging, owing to the difficulty of isolating specific defects (*e.g.*, boron vacancies and planar defects^12,17–20^) and the limited control over synthesis conditions.^21^

Atomistic simulations, in principle, could shed light on the relationships between specific compositional defects and mechanical properties of TMB_2_s, guiding the synthesis, micromechanical testing, and electron microscopy investigations. Density functional theory (DFT) calculations have provided basic insights, including predictions of 0 K elastic constants.^22^ Length scales accessible to DFT, however, do not allow for modeling more complex defects (being typically restricted to simple point defects) and loading scenarios. In particular, simulations of nanoindentation—the state-of-the-art tool for hardness measurements in TiB_2_ and ceramic thin films in general—are unfeasible for DFT. A suitable avenue for such simulations is molecular dynamics (MD), with the main limitation being the availability of reliable interatomic potentials. Only few empirical interatomic potentials exist for transition metal nitride/boride based ceramics, such as embedded atom method (EAM) potential for Ti–Al–N,^23^ MEAM (modified EAM) potential for Ti–N system with various stoichiometry,^24^ and 2NN-MEAM (second nearest-neighbor MEAM) potential for Ti–B.^25^ The last mentioned has been used to predict nano-hardness of defect-free TiB_2_ as a function of temperature (*T* = 0–1500 K) *via* nanoindentation simulations yielding 70 GPa at 0 K and 65 GPa at 300 K (≈10–20% overestimated compared with experiment). The rapidly developing filed of machine learning interatomic potentials (MLIPs)—promising DFT-level of accuracy and computational efficiency of (semi-)empirical potentials—has boosted MD simulations which are now becoming feasible for more materials of practical relevance.^26–28^ Recently, several MLIP training strategies suitable for nano-scale mechanical simulations of ceramics have been proposed,^29,30^ providing a basis for our work. While showing promise for reliable predictions of deformation and fracture mechanisms as well as quantities such as theoretical tensile strength and fracture toughness,^6^ these strategies have only been applied to ideal defect-free structures and need to be further tested/extended for simulations including highly defective atomic environments.

According to experimental findings, mechanical properties of TiB_2_ thin films vary significantly with B stoichiometry. For instance, the hardness of DC magnetron sputtered TiB_2−*x*_ films (with a B/Ti ratios 1.44–2.06) was reported to decrease from 43.9 GPa to 40.7 GPa, while the elastic modulus increased from 511 GPa to 541 GPa.^15,31^ Despite a substantial lack of boron, the hardness and Young's modulus are less affected. The mechanisms driving this phenomenon require detailed exploration. Based on the Ti–B phase diagram, some studies have treated TiB_2_ as a line compound,^32^ suggesting that under B-deficient conditions, the lack of B can be offset by formation of Ti-rich planar defects, locally alternating the crystalline structure and dislocation mobility. DFT calculated formation energy by Dahlqvist *et al.*^22^ suggested that B vacancies are energetically unfavorable, yielding ≈0.3 eV at^−1^ differences from the ideal stoichiometric TiB_2_ as B/Ti ratio vary from 1.5 to 2, whereas the formation of locally Ti-rich planar defects along the prismatic planes of TiB_2−*x*_ lattices is energetically more favorable than the formation of vacancies. These indications have been supported by transmission electron microscopy (TEM) investigations on various synthesized TiB_2−*x*_ thin films, showing that unpaired Ti atoms—resulting in B sub-stoichiometry—create planar defects on the prismatic plane of the TiB_2_.^1,17^

To elucidate how planar defects and randomly distributed boron vacancies impact the mechanical properties of boron-deficient TiB_2−*x*_ systems, in this work, we predict room-temperature mechanical properties of single-crystal TiB_2−*x*_ with various B contents realized *via* either point or planar defects, which were previously suggested to be largely present in magnetron-sputtered TiB_2_ coatings.^17,18,33^ Specifically, the elastic constants, hardness and indentation moduli are derived from nanoscale MD simulations of uniaxial tensile loading and nanoindentation. The underlying interatomic potential is machine-learned on various strained and/or defective *ab initio* bulk structures (active learning on configurations), up-fitted using highly extrapolative clusters representing environments near the indenter tip (on-the-fly training), and validated against *ab initio* calculations, and experimental data. The calculated mechanical properties as well as phenomena such as pop-in effects during nanoindentation align well with available experimental data. Further, the experimentally observed—and somewhat surprising—minor changes in hardness between the (nearly) ideal stoichiometric TiB_2_ and largely B-deficient TiB_2−*x*_, *x* ≈ 0.5, are attributed to Ti-rich planar defects. In a stark contrast, B vacancies cause much more significant hardness drop.

## 2 Methods

### 2.1 *Ab initio* datasets

*Ab initio* datasets for initial MLIP fitting, validation, and active learning were generated employing the Vienna *ab initio* Simulation Package (VASP^34–36^), together with the projector augmented wave (PAW) method and the Perdew–Burke–Ernzerhof exchange–correlation functional revised for solids.^34,37^ These included 0 K density functional theory (DFT) and finite-temperature Born-Oppenheimer *Ab initio* MD (AIMD) calculations^38^ using the Machine-Learning-Force-Field (MLFF) feature embedded in VASP. The on-the-fly learning in each AIMD run was initiated from scratch (with default parameters) and served only to accelerate generation of atomic environments for own MLIP training detailed in the following subsection. Testing the accuracy of total energies (forces, stresses, structural parameters) during VASP MLFF-aided runs against equivalent fully a*b initio* simulations yielded plausible accuracy. For illustration, the Root-Mean-Square-Error (RMSE) of total energies, atomic forces, and stresses were ≈1 meV per atom, 0.06 eV Å^−1^, 0.24 GPa, respectively. Regarding other parameters of our *Ab initio* calculations, the plane wave cut-off energy of 300 eV was applied together with the Γ point sampling of the reciprocal space.

The model of various TiB_2−*x*_ structures was based on a 720-atom (ideal α-TiB_2_, space group *P*6/*mmm*, Material ID:mp-1145)^39^ supercell, equilibrated at 300 K during simulations with the NpT (Isothermal–isobaric ensemble) ensemble (3 ps), followed by a simulation with the NVT (Canonical ensemble) ensemble (2.5 ps, using time-averaged lattice parameters from NpT).

To represent various off-stoichiometric environments, TiB_2−*x*_ structures with (i) randomly-distributed B vacancies, and (ii) uniformly distributed Ti-rich planar defects were simulated (2-step NpT → NVT equilibration at 300 K, similar as for TiB_2_). For MLIP training, we included 50% and 25% vacancy concentrations in the point defect structures. Ti-rich planar defects were constructed based on previous experimental observations.^17,18^ Specifically, these defects were created by removing boron atoms located between selected single or double (101̄0) prismatic Ti planes. These defect planes were oriented perpendicular to the [101̄0] crystallographic direction and uniformly distributed across the models. To generate environments representing various mechanical loading conditions, surfaces, and voids, tensile strains (2–10%; 30% “shock” tension inducing cleavage) and compressive strains (2% to 10%) were applied to selected TiB_2−*x*_ structures.

### 2.2 Machine-learning interatomic potential (MLIP) training

**Fig. 1:**
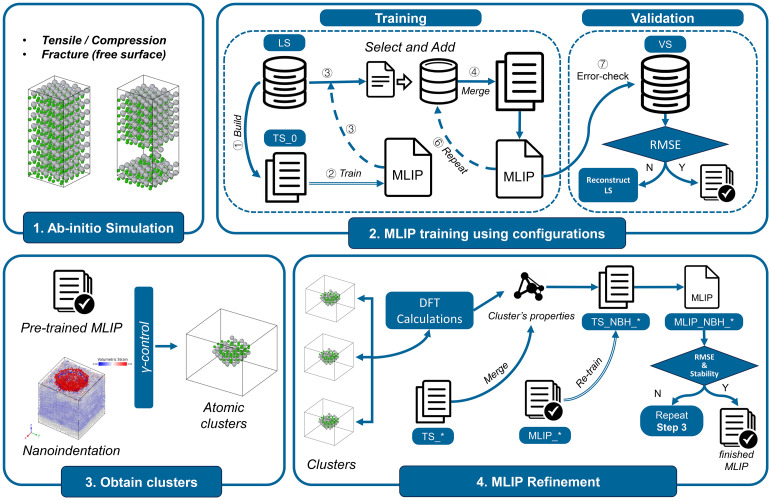
Schematic of our MLIP training routine. (1) Generation of initial training, learning, and validation set by finite-temperature *ab initio* MD calculations (point and planar defects, various strained structures). (2) Iterative active learning (on AIMD configurations) and validation using the concept of extrapolation grade, *γ*. (3) Preliminary nanoindentation runs to extract locally highly extrapolative environments (*γ* > *γ*_thr_) near the indenter tip, to be labeled by *ab initio* energies and forces in additional DFT calculations; (4) further MLIP re-training using atomic clusters from (3). Note that (3) and (4) represent on-the-fly training on environments near the indenter tip. In the schematic of atomic configurations gray and green spheres represent Ti and B atoms, respectively.

The mlip-3 package^40^—implementing the moment tensor potential (MTP) formalism—was used to train our MLIP, targeted to nanoscale mechanical simulations of (defective) AlB_2_-type TiB_2−*x*_ structures. The weights of energy and forces were set to 1 and 0.01, respectively, while stresses were not fitted due to the presence of atomic clusters in the training set. Our training procedure (Fig. 1) consisted of four main steps detailed below.

1. **Generation of AIMD dataset** (Fig. 1, Step 1). AIMD simulations (detailed in Sec. 2.1) were performed to generate a pool of *ab initio* configurations to be randomly split into the initial training set (TS_0_, 5%), the validation set (VS, 20%), and the learning set (LS, the rest ≈75%).

2. **Active learning on AIMD configurations** (Fig. 1, Step 2). The initial MLIP_0_ was trained on TS_0_ using an untrained 16-level MTP. Consequently, the MaxVol algorithm^41,42^ was employed to identify extrapolative configurations in the LS to be added to the TS based on their extrapolation grade (*γ*). In simple words, *γ* is the “uncertainty indicator” quantifying the degree to which an atomic configuration deviates from local environments in the training set. Higher *γ* indicates higher extrapolation into unfamiliar configuration space where energy (interatomic force, stress) predictions may no longer be reliable. From the definition (eqn (9) in ref. 42), *γ* ≤ 1 and *γ* > 1 imply MLIP's interpolation and extrapolation, respectively, and *γ*_thr_ = 2 has been previously used as *accurate* extrapolation.^29,42,43^ In this stage, a threshold *γ*_thr_ = 2 is set for active learning. The final MLIP produced in Step 2 exhibited *γ* ≤ 1 for the TS, *γ* < 2 for the LS but also the—independent, unseen—VS. Training and validation errors for energies and forces, as quantified by Root-Mean-Square-Error (RMSE), were below 6 meV per atom and 0.25 eV Å^−1^.

3. **Preliminary nanoindentation simulations to identify extrapolative environments near the indenter tip** (Fig. 1, Step 3). Using the MLIP from Step 2, we simulate nanoindentation tests (for computational details see Sec. 2.2.1) and output the per-atom *γ* value at each step. These allow identifying locally highly extrapolative environments (*γ* ≫ 10 in a spherical atomic region with a 9 Å radius). The clusters—embedded in a box with a 10 Å vacuum buffer—were labeled by *ab initio* energies and forces in additional DFT calculations (see Sec. 2.1).

4. **MLIP refinement using extrapolative atomic clusters** (Fig. 1, Step 4). Atomic clusters generated above were added to the TS and the MLIP was re-trained until no highly extrapolative environments (*γ* > 10) were present during MD nanoindentation test for both the ideal stoichiometric TiB_2_ as well as B-substoichiometric structures (on-the-fly training). MLIP's transferability to various TiB_2−*x*_ structural variants (some of which were directly trained on already in Step 2) is further discussed in the Results section.

#### 2.2.1 Molecular dynamics (MD) simulations

MD simulations were performed using the Large-scale Atomic/Molecular Massively Parallel Simulator (LAMMPS)^44,45^ interfaced with the mlip-3 package (requiring custom compilation) to enable the use of MTP-type machine-learning force fields. The force field was trained by us, as detailed in the previous section. All simulations used the extrapolation control (*γ*, explained in the previous section) to monitor the reliability of the underlying potential. Specifically, *γ* = 10 was set for terminating the simulation, but this critical value has never been reached during mechanical tests presented in the Results section. The timestep was always set to 1 fs. The Open Visualization Tool software (OVITO^46^) served to visualize and analyze the results.

##### TiB_2−x_ models

**Fig. 2:**
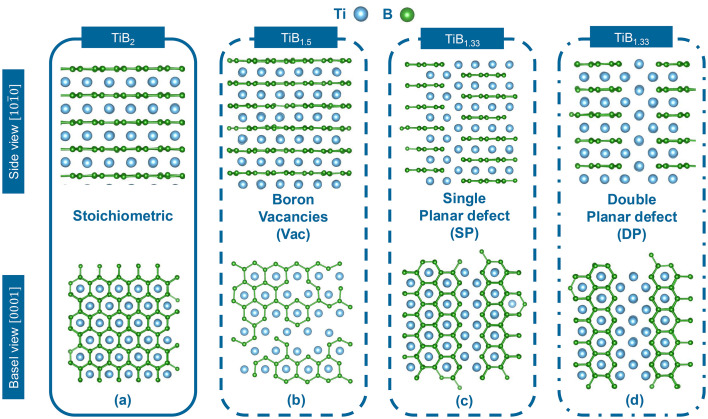
Illustration of our B-deficient TiB_2−*x*_ models. (a) The ideal stoichiometric TiB_2_, (b) TiB_2−*x*_, *x* = {0.2; 0.3; 0.4; 0.5}, with randomly distributed B vacancies also referred to as Vac; (c) TiB_2−*x*_, *x* = {0.67}, with single Ti-rich planar defect also referred to as SP; (d) TiB_2−*x*_, *x* = {0.67}, with double Ti-rich planar defect also referred to as DP. The visualization is at the (101̄0) plane (top row) and the basal (0001) plane (bottom row), respectively, generated using the program VESTA.^47^

The ideal AlB_2_-type α-TiB_2_ was modeled in a 120 000-atom supercell (≈11 × 11 × 11 nm^3^). B-substoichiometric structures were considered *via* (i) randomly distributed B vacancies, or (ii) uniformly distributed planar defects, specifically, Ti-rich planar defects observed in experiment^17,18^ and visualized in Fig. 2. All structures were fully equilibrated at 300 K during a 10 ps NpT simulation.

##### Mechanical tests with fully periodic boundary conditions

For selected systems (TiB_2_, TiB_1.8_, TiB_1.7_, TiB_1.6_, and TiB_1.5_), room-temperature uniaxial tensile and shear tests were simulated by applying strain along the [0001], [101̄0], and [1̄21̄0] direction or along the (0001)[101̄0] slip system, respectively. The strain rate was 0.5 m s^−1^ and the simulation was continued until fracture (tensile test) or lattice slip (shear test). Tensile tests considered Poisson's contraction (*via* the NpT ensemble orthogonal to the loading direction), while shear test were conducted at a constant volume. Stress/strain data from the elastic region (strain ≤4%) served to derive the second-order elastic constants, *C*_*ij*_. Based on *C*_*ij*_s, we evaluated elastic moduli (*e.g.*, the bulk, Young's, and shear modulus) following standard formulas.^48^

##### Nanoindentation simulations for MLIP training (detailed in Sec. 2.2)

To identify environments representing highly strained regions near the indenter tip in an efficient manner, nanoindentation tests were performed in medium-sized supercells (≈6000 atoms, 3.5 × 3.5 × 3.5 nm^3^) at a high loading rate (≈200 m s^−1^), considering a spherical indenter with a 1 nm radius. If present, highly extrapolative environments (with *γ* ≫ 10) were extracted from each simulation step and used to re-train our MLIP.

##### Nanoindentation simulations for quantification of mechanical properties

**Fig. 3:**
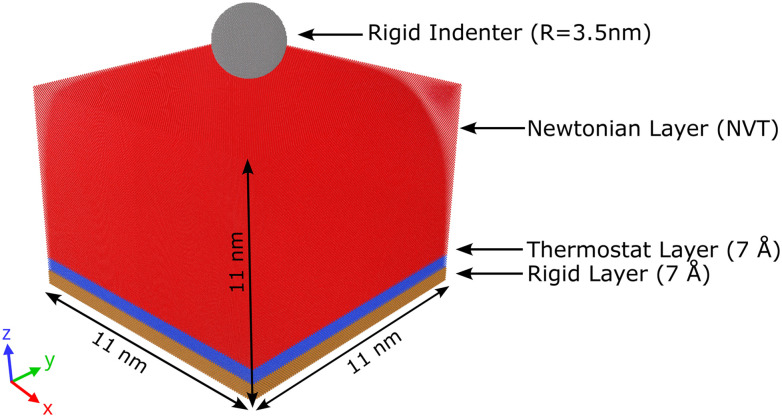
Schematic of our nanoindentation model. Atoms in the simulation box were grouped into (i) a 7 Å thick rigid layer with frozen atoms to prevent movement along the *z* axis, (ii) a 7 Å thick thermostat layer in which the temperatures fixed at 300 K using temperature scaling thermostat, and (iii) a Newtonian layer.

The model (Fig. 3) included a TiB_2−*x*_ supercell (≈11 × 11 × 11 nm^3^) together with a virtual repulsive indenter. Periodic boundary conditions (PBCs) were considered along the *x*‖[1̄21̄0] and *y*‖[101̄0] directions, while *z*‖[0001] was non-periodic: with lower and upper boundary being fixed and shrink-wrapped, respectively, to ensure that all atoms remain within the simulation box.

Prior to nanoindentation, the simulation box was equilibrated at the target temperature (*T* = 300 K for most simulations) for 10 ps using NpT ensemble, followed by a 2.5 ps simulation with in NVT ensemble with Nosé–Hoover thermostat. Nanoindentation was simulated in a displacement-controlled mode, by moving a virtual indenter (3.5 nm radius, force constant *K* = 10 eV Å^−3^) along the [0001] axis for 140 ps at loading speed of 15 m s^−1^, until reaching ≈20 Å indentation depth. The hardness was evaluated from the hardness result averaged at the indentation depth of (15 ± 0.5) Å, corresponding to approximately 15% of the film thickness, in order to minimize the influence of the infinitive hard substrate on the hardness measurement. After holding the indenter for 2 ps, unloading was simulated for another 100 ps.

The hardness, *H*, was evaluated following Oliver–Pharr method,^21,49^*i.e.*, from the relationship between the indentation load, *P*, and the projected contact area, *A*, during indentation process,1
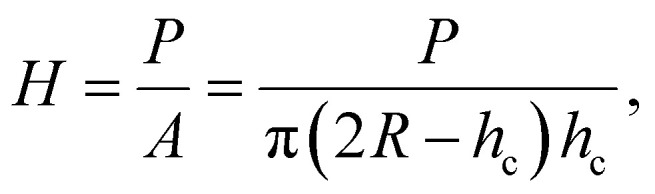
where *R* is the (spherical) indenter radius and *h*_c_ is the current projected contact depth. The reduced elastic modulus, *E*_r_, was derived from the unload phase as2
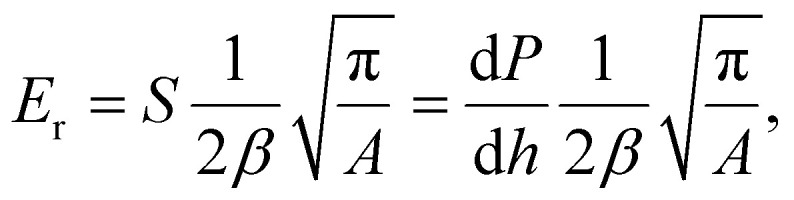
where *S* = d*P*/d*h* is the stiffness (extracted by fitting a linear function using the linear elastic interval of load–displacement data during the unload) and *β* = 1 is the correction factor. Elastic deformation is generally observed in both indented material and indenter tip during the indentation process. The relationship between the reduced elastic modulus (*E*_r_), the elastic modulus of the indented material (*E*_s_), and that of the indenter tip (*E*_i_) is given by:3
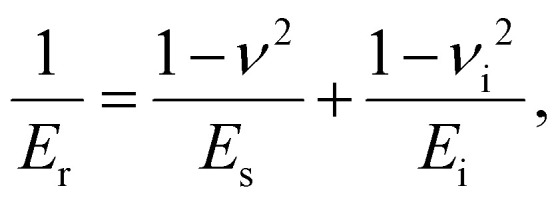
where *ν* is the Poisson's ratio of the indented material and *ν*_i_ the indenter. For an ideal virtual infinitely rigid indenter, *E*_i_ = +∞, thus, eqn (3) simplifies as^50^4
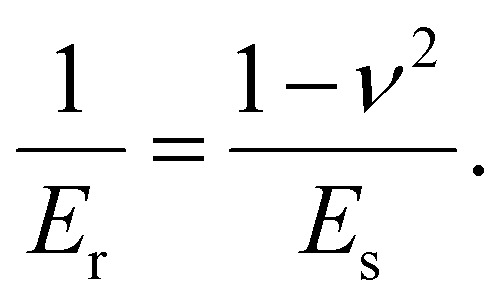


In this work, Poisson's ratios for the investigated material systems were obtained from elastic constants, *C*_*ij*_s, calculations and are listed in Table 2.

## 3 Results and discussion

### 3.1 Validation of the trained MLIP

To carry out nanoscale MD nanoindentation tests for various defective TiB_2−*x*_ structures, we trained machine learning interatomic potential (MLIP) based on moment tensor potentials (MTP) formalism, building on previous training routine developed for tensile and shear simulations of single-crystal ceramics.^29,43^ Enabling accurate description of complex, highly strained regions near the indenter tip, this routine was essentially upgraded by on-the-fly training on extrapolative atomic clusters from simplified nanoindentation models using a pre-trained MLIP (for details see the Methods and Fig. 1). Prior to employing the trained MLIP, we show its capability to reproduce material properties consistently with 0 K and finite-temperature *ab initio* results, as well as available experimental data.

**Fig. 4:**
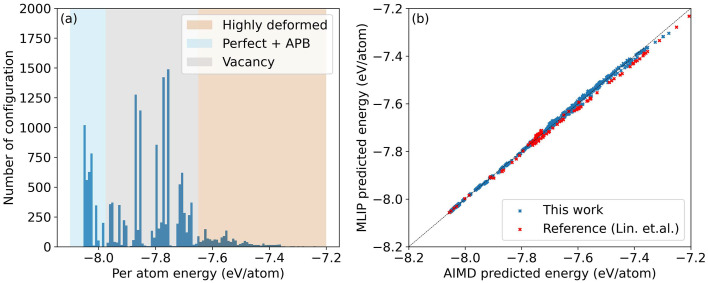
Validation of the here-trained MLIP against a reference finite-temperature *ab initio* dataset (≈10^4^ configurations). (a) The total energy distribution in the reference set containing various defective and/or highly-strained TiB_2−*x*_ configurations, as described in the Methods. The B off-stoichiometry is realized *via* randomly distributed vacancies or planar defects (Vac, SP, DP–see Fig. 2). (b) Linear correlation between the corresponding AIMD and MLIP-predicted energies (blue data points) together with values predicted for another reference dataset from ref. 29 containing snapshots from finite-temperature (300 and 1200 K) AIMD simulations of TiB_2_ subject to uniaxial tensile and shear loading until fracture or lattice slip.

Validation against an independent *ab initio* dataset—that contains ≈10^4^ snapshots from finite-temperature AIMD simulations of TiB_2−*x*_ featuring a wide range of point and planar defect types/concentrations/distributions as well as these structures under tensile, shear, or compressive loads—supports the credibility of our MLIP (Fig. 4). Fig. 4a depicts the histogram of total energies in the reference set, while the corresponding parity plot is given in Fig. 4b. The AIMD *vs.* MLIP-predicted energies fall at or near the *x* = *y* line (Fig. 4b), showing that the model closely reproduces *ab initio* values, as quantified also by statistical errors RMSE = 6 meV and *R*^2^ = 0.999. Additional comparison with the *ab initio* MD dataset (300 and 1200 K data) from our previous work^29^ yields RMSE and *R*^2^ values of 13 meV and 0.999, respectively.

Furthermore, our MLIP accurately reproduces stress/strain curves and failure mechanisms derived from room-temperature tensile and shear tests for defect-free TiB_2_ in ref. 29, in particular, yielding accuracy in stresses of <1 GPa where slightly higher deviations from AIMD data may occur near the fracture point. To assess the MLIP's transferability beyond DFT accessible length scales and point/planar defect concentrations and configurations not directly trained on, we simulate room-temperature equilibration of TiB_2_ as well as newly generated TiB_2−*x*_ structures (*e.g.*, TiB_1.6_, TiB_1.8_), employing supercells with ≈11 000 atoms (*i.e.*, beyond DFT accessible length scales). Comparison of the calculated lattice parameters (Table 1) with reference *ab initio* or experimental values shows excellent agreement. Specifically, the differences are 0.02 Å (0.6%) and 0.006 eV at^−1^ (0.07%) in terms of lattice parameters and energies, respectively. Importantly, the MLIP's extrapolation grade during all equilibrations remains within the *reliable* extrapolation regime (*γ* < 10 (ref. 29 and 51)).

**Table 1:** Validation of the here-trained MLIP in terms of structural parameters of various TiB_2−*x*_ structures. Comparison of time-averaged lattice parameters (*a* and *c* in Å) obtained by MLIP-MD and equivalent *ab initio* MD simulations at temperature *T* = 300 K, or experimental values. Defects are categorized as vacancies (Vac) and planar defects (SP, DP), as schematically depicted in Fig. 2

Structure	Defect type	Method	*T*	*a*	*c*
	—	*ab initio* - MD	300	3.037	3.218
		MLIP-MD	300	3.040	3.236
		DFT^29^	0	3.027	3.213
TiB_2_		DFT^52^	0	3.028	3.226
		Experiment^53^	300	3.030	3.230
		Experiment^54^	300	3.030	3.240
TiB_1.8_	Vac	*ab initio* – MD	300	3.048	3.183
		MLIP-MD	300	3.049	3.197
TiB_1.7_	Vac	*ab initio* – MD	300	3.055	3.155
		MLIP-MD	300	3.054	3.179
TiB_1.6_	Vac	*ab initio* – MD	300	3.058	3.139
		MLIP-MD	300	3.056	3.154
TiB_1.5_	Vac	*ab initio* – MD	300	3.059	3.137
		MLIP-MD	300	3.061	3.132
TiB_1.33_	SP	*ab initio* – MD	300	3.046	3.259
		MLIP-MD	300	3.056	3.261
TiB_1.33_	DP	*ab initio* – MD	300	3.034	3.236
		MLIP-MD	300	3.055	3.251

**Fig. 5:**
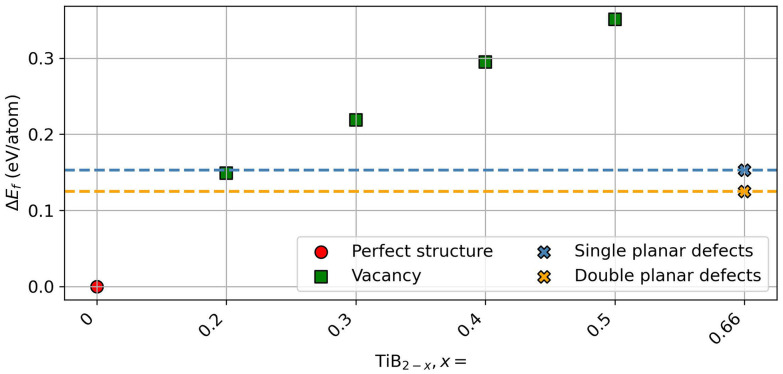
Formation energy difference (Δ*E*_f_) between various B-substoichiometric TiB_2−*x*_ (*x* = 0.2, 0.3, 0.4, 0.5, 0.66) structures calculated at 0 K using the here-trained MLIP. Defects are categorized as vacancies (green square), planar defects (SP: blue cross, DP: yellow cross), as schematically depicted in Fig. 2. Note that the MLIP-predicted *E*_f_ values differ from own reference DFT calculations by less than 0.01 eV at^−1^, underpinning the credibility of our force field.

Using the here-trained MLIP, we have additionally calculated 0 K formation energies, *E*_f_, of representative non-stoichiometric TiB_2−*x*_ structures (Fig. 2 and Fig. 5). The results indicate that all defects cost energy (Δ*E*_f_ > 0 considering TiB_2_ as a reference state) and TiB_2−*x*_ variants with disordered vacancies are significantly more expensive than their chemically equivalent counterparts containing single- or double-planar defects (SP, DP). These predictions are consistent with previous 0 K DFT indications^22^ and experimental (electron microscopy) findings.^17,18^ The defects considered here are those frequently mentioned in experimental investigations of diborides. While there are other ways to realize B substoichiometry—such as Ti substitutional defects, Ti interstitials, or planar defects at different planes—none of those has been evidenced in experiment is therefore excluded from our models.

In what follows we present MD simulations—particularly nanoindentation tests for various off-stoichiometric TiB_2−*x*_ systems—that are beyond length scales accessible to *ab initio* calculations. Their reliability is inferred from (i) the previously presented MLIP's validation against *ab initio* data, and (ii) MLIP's extrapolation grades, *γ* (see the Methods), monitored during some of these simulations. Importantly, *γ* values indicate *reliable* extrapolation (*γ* < 10) even under far-from-equilibrium conditions characterized by high stresses, local distortions, Ti/B intermixing, and crack nucleation beneath the indenter tip. MLIP's reliability at the nanoscale is further supported by comparing the calculated material's properties, such as elastic constants and hardness, with reference DFT or experimental data (see following sections).

### 3.2 Elastic constants, tensile and shear strength of defective TiB_2−*x*_

Employing the here-trained MLIP, we simulate tensile and shear deformation of various defective TiB_2−*x*_ structures, considering low-index crystallographic directions and slip systems. The simulations are carried out at 300 K, for supercells with ≈110 000–150 000 atoms, under fully periodic boundary conditions. Prior to deformation, all structures are fully relaxed, exhibiting excellent thermal stability. In particular, no B diffusion is observed even for largely off-stoichiometric models (*e.g.* TiB_1.6_), mirroring the intrinsic structural stability of the α phase which imposes high energy barrier for vacancy migration and diffusion. According to the Arrhenius relationship, this results in diffusion at 300 K unlikely, making macroscopic observation virtually impossible.^55–57^

**Fig. 6:**
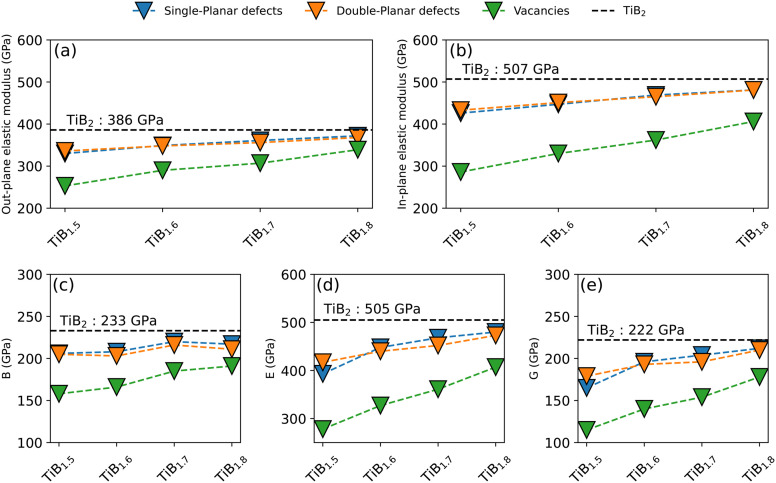
Room-temperature directional Young's moduli, polycrystalline Young's modulus, bulk modulus and shear modulus of the ideal TiB_2_ as well as of various B-substoichiometric TiB_2−*x*_ structures derived using the here-trained MLIP. (a) in-plane Young's modulus *E*_[1̄01̄0]_ = *E*_[1̄21̄0]_; (b) out-of-plane Young's modulus *E*_[0001]_; (c) bulk modulus, *B*; (d) Young's modulus, *E*; and (e) shear modulus, *G* were obtained from the elastic constants, *C*_*ij*_. The B deficiency in TiB_2−*x*_ was realized by uniformly distributed single- or double-planar defects (SP: blue data points, DP: orange data points), or disordered vacancies (Vac: green data points), as schematically depicted in Fig. 2. The dashed horizontal line marks the ideal TiB_2_. For the underlying elastic constants and numerical values of the elastic moduli, see Table 2.

The linearly-elastic part of stress/strain curves (Fig. S1 in SI) serves to derive second-order elastic constants, *C*_*ij*_, and related mechanical properties (Table 2, Fig. 6). Furthermore, global stress maxima in the stress/strain data are used as estimates of the theoretical tensile and shear strength (Table S1 in SI) following ref. 58 and 59.

**Table 2:** Room-temperature mechanical properties of the ideal TiB_2_ as well as of various B-substoichiometric TiB_2−*x*_ structures derived from uniaxial tensile and shear tests at 300 K using the here-trained MLIP. Second-order elastic constants, *C*_*ij*_ (in GPa), were obtained from the linearly-elastic part of the stress/strain curves, and were subsequently used to calculate the polycrystalline bulk, shear, and Young's modulus (*B*, *G*, *E* in GPa) and the Poisson's ratio (*ν*). The B deficiency in TiB_2−*x*_ was realized by uniformly distributed single- or double-planar defects (SP, DP), or disordered vacancies (Vac), as schematically depicted in Fig. 2

Models	Defect type	*T*	*C*_11_	*C*_12_	*C*_13_	*C*_33_	*C*_44_	*B*	*G*	*E*	*ν*
TiB_1.5_	SP	300	461	86	100	336	156	206	165	392	0.183
	DP		466	78	98	371	183	205	179	417	0.161
	Vac		319	73	85	291	113	157	115	278	0.206
TiB_1.6_	SP	300	478	83	94	381	217	208	195	447	0.142
	DP		478	74	89	377	204	203	193	440	0.140
	Vac		357	70	78	319	143	165	139	326	0.171
TiB_1.7_	SP	300	503	86	103	397	224	220	204	467	0.146
	DP		497	79	101	392	205	216	196	452	0.151
	Vac		395	79	92	334	162	184	153	361	0.174
TiB_1.8_	SP	300	510	84	92	401	236	216	212	480	0.131
	DP		506	67	90	397	225	211	210	473	0.126
	Vac		434	76	83	366	191	191	177	406	0.145
TiB_2_	—	300	542	89	105	421	246	233	222	505	0.138
TiB_2_ ^48^	—	0	670	64	101	473	267	263	269	601	0.119
TiB_2_ ^29^	—	300	588	85	98	409	261	236	246	554	0.113
TiB_2_ ^60^	—	300	654	56	98	454	263	250	262	583	0.111

Fig. 6 underscore the strong influence of the B content on TiB_2_'s mechanical properties already in the linearly-elastic regime. Specifically, the in-plane Young's modulus, *E*_[1̄21̄0]_ (Fig. 6a), decreases from 507 GPa to 426 GPa (16% decrease) when going from TiB_2_ to TiB_1.5_, where the latter contains planar defects (SP, DP). Due to the prismatic plane isotropy, differences between *E*_[1̄21̄0]_ and *E*_[101̄0]_ are negligible. The out-of-plane Young's modulus, *E*_[0001]_ (Fig. 6b), drops from 386 GPa (TiB_2_) down to 330 GPa (TiB_1.5_, 15% decrease) with planar defects. While the B-sub-stoichiometry-mediated decrease in directional Young's moduli is slightly more pronounced for single planar defects (SP), the differences from double planar defects (DP) remain rather small (<10 GPa). Compared with planar defects, the directional Young's modulus of TiB_2−*x*_ containing disordered vacancies (Vac) is significantly lower. Trends in the polycrystalline *B*, *G*, and *E* (Fig. 6c–e) follow these observed for the directional Young's modulus, in particular, decrease as the *B* sub-lattice becomes less populated. This decrease is the least pronounced for the bulk modulus.

The polycrystalline Poisson's ratio (*ν*, shown in Table 2) increases from 0.14 (TiB_2_) up to 0.16–0.2 (TiB_1.5_ with planar defects or vacancies), indicating an improved ability to redistribute stresses *via* plastic deformation, as discussed later in relation to the results obtained from nanoindentation simulation. A Poisson's ratio (*ν*) of 0.25 has been previously assumed when deriving nanoindentation hardness in experimental studies.^61^ This may, however, not accurately reflect the actual TiB_2_‘s stoichiometry, causing discrepancies in the literature. When deriving Young's modulus from our later presented nanoindentation tests, we therefore use Poisson's ratios representing the correct stoichiometry and defect type (*i.e.*, SP, DP, or Vac). Using a constant *ν* = 0.25, the values would change by up to 5%.

**Fig. 7:**
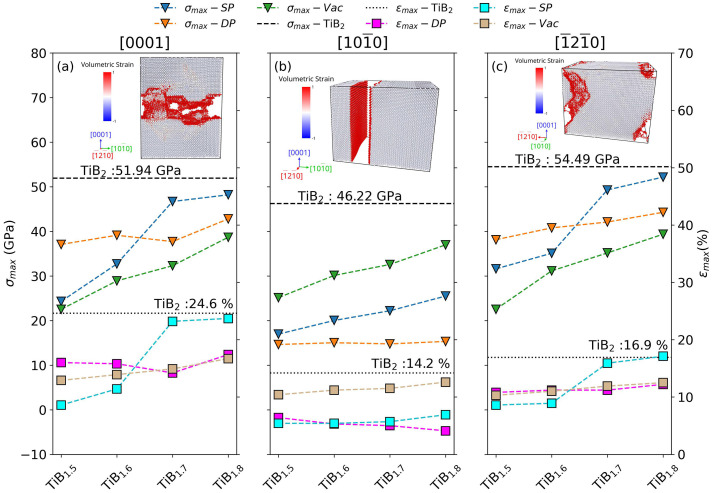
Theoretical tensile strength (and the corresponding fracture strain) of the ideal TiB_2_ as well as of various B-substoichiometric TiB_2−*x*_ structures derived using the here-trained MLIP. (a), (b) and (c) depict the tensile strength (*σ*_max_; triangle data points, left *y*-axis) and fracture strain (*σ*_max_; square data points, right *y*-axis) along the [0001], [101̄0] and [1̄21̄0] crystallographic directions, respectively. The B deficiency in TiB_2−*x*_ was realized by uniformly distributed single- or double-planar defects (SP, DP), or disordered vacancies (Vac), as schematically depicted in Fig. 2. The *σ*_max_ values of SP, DP, Vac are shown in blue, orange and green lines, respectively. The *ε*_max_ of SP, DP and Vac are shown in cyan, magenta and brown lines, respectively. The structure snapshots (color code based on the volumetric strain) show TiB_1.8_ with SP near the fracture point, illustrating the fracture pattern. These general fracture patterns are similar for other stoichiometries and defect types.

Furthermore, Fig. 7 depicts trends in the ideal strengths, *σ*_*hkil*_, of TiB_2−*x*_, derived from the maximum stress withstood during our room-temperature uniaxial tensile simulations. Irrespective of the defect type (SP, DP, Vac), our predictions reveal *σ*_[101̄0]_ < *σ*_[0001]_ < *σ*_[1̄21̄0]_, suggesting that the basal plane isotropy does not extend beyond linearly-elastic regime. For the corresponding fracture strains, *ε*^F^_[*hkil*]_, we predict *ε*^F^_[101̄0]_ < *ε*^F^_[1̄21̄0]_ < *ε*^F^_[0001]_. Although theoretical tensile strengths generally diminish with decreasing the B content, the trends seem more conspicuous than those derived from elastic constants. In particular, TiB_1.7_ with single planar defects exhibits the second highest strengths—after the ideal stoichiometric TiB_2_—followed by TiB_1.7_ with double planar defects, which, however is notably below, by >20%. Increasing the fraction of double planar defects, tensile strength of TiB_1.5_ remains essentially unchanged compared with TiB_1.7_, while increasing the fraction of single planar defects, TiB_1.5_, yields the overall lowest strength.

The results in Fig. 7 also suggest that both types of planar defects deteriorate tensile strength parallel to the Ti-rich defect region. Analysis of [101̄0] tensile tests reveals cleavage along the entire Ti-rich plane (structure snapshots in Fig. 7). Possible explanation is that the Ti–Ti bond strength within the Ti-rich region, perpendicular to the planar defect, is weaker than the intact hexagonal structure with not only Ti–Ti bonds but also Ti–B bonds, in stoichiometric TiB_2_. This differences in distribution of mechanical properties throughout the simulation box increases stress concentration at the Ti-rich plane, causing cleavage fracture along these planes.

**Fig. 8:**
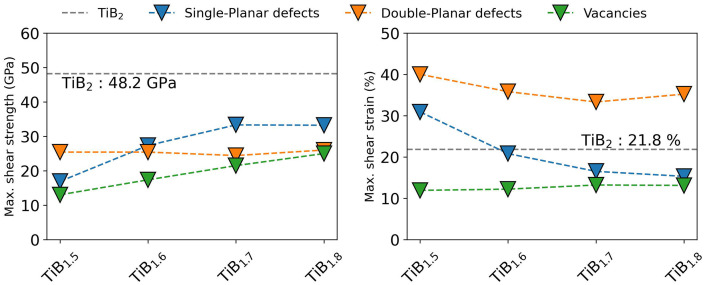
Room-temperature shear strength of TiB_2−*x*_ systems derived from (0001)[101̄0] shear tests using the here-trained MLIP. (a) Shear strength and (b) the corresponding slip strain. Results for the ideal TiB_2_ and defective TiB_2−*x*_ structures—single-planar defects, double-planar defects, and randomly distributed B vacancies (schematically depicted in Fig. 2)—are marked in blue, orange and green, respectively. The underlying values are shown in Table S1 in SI.

Furthermore, considering the typical orientation of diboride thin films—with (0001) planes perpendicular to the growth direction^62^—we simulate shearing along the (0001)[101̄0] slip system. These are also representative of local stress states during nanoindentation. Fig. 8 presents the theoretical shear strength (together with the corresponding slip strain) derived as the maximum stress withstood by the material during shear deformation. The ideal TiB_2_ exhibits the highest shear strength but only an intermediate fracture strain compared with the B-deficient systems. Consistently with the shear modulus trends in Fig. 6, TiB_2−*x*_ with disordered vacancies show a linear shear strength increase from ≈10 to 20 GPa. The correlation of the corresponding slip strain and B content, however, is rather weak. For single-planar defects, the shear strength initially increases with the B content, reaching a plateau for TiB_1.7_. The corresponding slip strain decreases significantly, as the B/Ti ratio decrease. For double-planar defects, the shear strength is the highest and remains stable despite stoichiometry changes, which can be attributed to a more favorable Ti–B bonding environment and improved p–d orbital hybridization, as shown in the SI (Fig. S3). The defective layers also help distribute the applied shear over a wider region, reducing local stress concentration. As a result, the reduction in slip strain is more moderate compared with the SP case.

Overall, simulations of tensile and shear tests indicate that the mechanical properties of TiB_2−*x*_ are controlled by both the B content and the underlying defect type. In some experimental observations, however, sub-stoichiometric TiB_2−*x*_ thin films—somewhat surprisingly—were reported to possess hardness as high as the stoichiometric TiB_2_.^31,33^ To further investigate this discrepancy, nanoindentation simulations are carried out in the following section.

### 3.3 Nanoindentation of defective TiB_2−*x*_

To shed light on mechanisms driving the variation in hardness and Young's modulus of perfect TiB_2_ and sub-stoichiometric TiB_2−*x*_ thin films, room-temperature MLIP-MD nanoindentation simulations for both ideal stoichiometric as well as B-sub-stoichiometric structures were performed. In line with most TiB_2_ (and other transition metal diboride) films being preferentially [0001] oriented during the synthesis,^1,2,63^ our model considers an indenter moving along the [0001] crystallographic axis.^3^ Detailed description of the simulation setup and derivation of the corresponding hardness and Young's modulus values is given in the Methods.

**Fig. 9:**
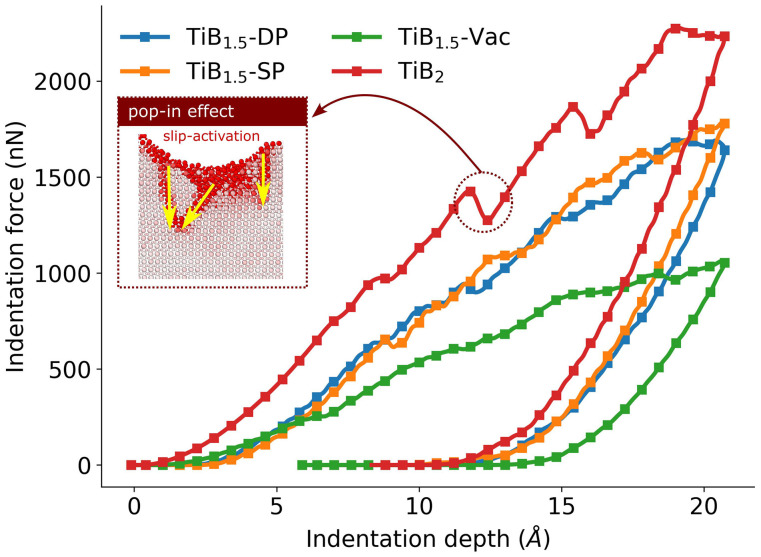
Representative load–depth (L–D) curves derived from room-temperature nanoindentation simulations for TiB_2_ as well as various B-substoichiometric TiB_2−*x*_ structures. These nanoscale simulations were carried out using the here-trained MLIP. The B deficiency in TiB_2−*x*_ was realized by uniformly distributed single- or double-planar defects (SP: orange data points, DP: blue data points), or disordered vacancies (Vac: green data points), as schematically depicted in Fig. 2.

Fig. 9 presents the obtained load-depth or load–displacement (L–D) curves for the ideal TiB_2_ as well as its defective TiB_2−*x*_ counterparts, represented by TiB_1.5_, in which the B-sub-stoichiometry is realized by planar defects (SP, DP) or B vacancies (Vac). Irrespective of the B content, pressing the indenter into the (0001) surface initially induces an elastic response. As the indentation depth (*h*_c_) increases, the reactive force on the indenter exhibits a small bump indicating the “pop-in” effect, hence hinting at the onset of plastic deformation *via* dislocation motion. These observations closely align with L–D curves obtained from lab-scale nanoindentation tests.^64,65^ The pop-in event observed in the load–displacement curve is primarily due to the sudden activation of specific slip planes (mainly basal and pyramidal planes) beneath the indenter tip.^66^ These “pop-in” effects seem to reflect the B vacancy distribution, in particular, are notable for structures containing planar defects—which can be viewed as highly ordered vacancy arrangement—but almost negligible for those with vacancies randomly distributed in the B network.

**Fig. 10:**
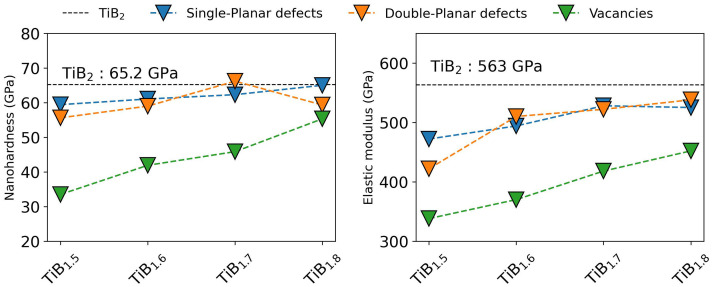
The hardness (a) and Young's modulus (b) derived from room-temperature nanoindentation simulations using the here-trained MLIP. The results for TiB_2_ (the dashed horizontal line) are compared with various B-substoichiometric TiB_2−*x*_ structures. The SP and DP refer to single and double-planar defects, respectively, as schematically depicted in Fig. 2.

The calculated L–D data was further used to determine the hardness, *H*, and Young's modulus, *E* (Fig. 10). With *H* = 65 GPa and *E* = 563 GPa, TiB_2_ ranks as the second highest and the highest in terms of *H* and *E*, respectively, which is ≈10–20% overestimated with respect to experiment. Such discrepancy can be ascribed to the fact that real samples are never perfectly pure and ideally defect-free. Besides simple point and planar defects considered in our work, *H*(*E*) measurements are influenced by other macroscopic features like grain boundaries, micro-cracks as well as porosity arising from various deposition conditions. As previously shown^67–71^ the yield strength, thus hardness of ceramics, is dependent on the grain size following the Hall–Petch relationship. Quantitative assessment of these effects for TiB_2_, however, is outside of our scope.

While both the predicted *H* and *E* decrease upon decreasing the B content, *i.e.*, by introducing more defects, there is an apparent difference between effects of B vacancies and planar defects, where the former cause a more dramatic hardness and Young's modulus deterioration. For structures with B vacancies, *H* and *E* decrease steadily by 49% and 40%, respectively, when the Ti-to-B ratio changes as 1 : 2 → 1 : 1.5, *i.e.*, when 25% of the B sub-lattice becomes unpopulated. The *H* and *E* decrease in TiB_2−*x*_ with planar defects occurs at a significantly slower rate. In the presence of single-planar defects, *H* = 59–62 GPa (TiB_1.5_ → TiB_1.8_), which is only a 10% to 5% decrease compared with the ideal TiB_2_, thus, the effect of decreasing the B content massively—down to TiB_1.5_ —causes only minimal H changes. For TiB_2−*x*_ with double-planar defects, *H* = 66 GPa of TiB_1.7_ even slightly exceeds that of the defect-free TiB_2_.

Our simulations (Fig. 10b) further indicate that the Young's modulus *E* = 563 GPa of TiB_2_ decreases to 338 GPa (B vacancies) or 472 GPa (SP) or 422 GPa (DP) for the TiB_1.5_ stoichiometry. The differences between SP and DP defects are generally minor, especially compared with the significant E decrease in vacancy-containing TiB_2−*x*_ structures. Note that B vacancies are more detrimental not only to TiB_2_'s mechanical performance but also its energetic stability, as suggested by us as well as by previous DFT formation energy calculations.^22^ These simulations help to understand recent experimental findings^33^ demonstrating that the hardness of TiB_2−*x*_ thin films deposited *via* Direct Current Magnetron Sputtering (DCMS) is maintained or slightly enhanced upon reducing the B/Ti ratio from 2.12 to 1.46, whereas the elastic modulus exhibits a noticeable decline. In particular, according to our predictions, the hardness remains nearly constant or undergoes a slight reduction as the B/Ti ratio decreases from 2 to 1.7 and eventually to 1.5. Conversely, the Young's modulus decreases significantly as the B/Ti ratio varies from 2 to 1.5, particularly in models with SP and DP defects.

**Fig. 11:**
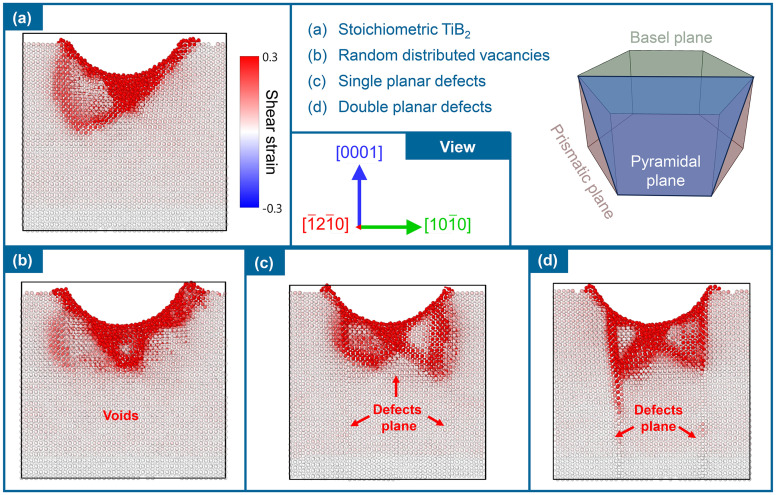
Atomic shear strain distribution at the final stage of nanoindentation tests simulated with the here-trained MLIP. (a) Stoichiometric TiB_2_, (b–d) off-stoichiometric TiB_1.8_ with disordered vacancies (b), single planar defects (c), and double planar defects (d), as schematically depicted in Fig. 2. The color code is mapped using the atomic strain analysis function in OVITO.^46^ The supercell slice has thickness of ≈1 nm, oriented perpendicular to the prismatic plane. The tripod in color (top right) indicates crystallographic directions.

To develop basic level of understanding why disordered B vacancies and Ti-rich planar defects trigger a distinct mechanical response, Fig. 11 shows the volumetric shear strain distribution in the indented models in which the indenter is pressed fairly deep in the sample (indentation depth ≈2 nm). The analysis indicates qualitatively different stress redistribution due to the presence of planar defects compared with disordered B vacancies as well as the ideal TiB_2_. In particular, TiB_2−*x*_ structure with B vacancies reveals highly concentrated shear strain below the indenter tip and fewer slip events, in contrast to the defect-free TiB_2_ as well as TiB_2−*x*_ featuring Ti-rich planar defects. This may diminish the occurrence of “pop-in” effects in models with B vacancies. Unlike that, TiB_2−*x*_ with planar defects (both SP and DP) restricts lattice slip between several planar defects beneath the indentation zone. These indications are consistent with Thörnberg *et al.*,^31^ suggesting that Ti-rich planar defects act as barriers to dislocation glide, contributing to anisotropic deformation behavior and high hardness.

## 4 Conclusion and outlooks

Employing MLIP-MD simulations, we predicted fundamentally different mechanical response of a paradigm hard coating material, TiB_2_, depending on (i) the B content—within the experimentally reported B/Ti ratios of 2 to 1.5—and, (ii) point *vs.* planar defects realizing the same stoichiometry. The defect types were motivated by other DFT studies (vacancies) and experimental high-resolution electron microscopy investigations (Ti-rich single and double planar defects). Our machine-learning force field (MTP-type MLIP) for finite-temperature nanoindentation simulations was trained by active learning on both stoichiometric and off-stoichiometric TiB_2−*x*_ structures subject to tensile, shear, or compressive loads, as well as on-the-fly (OTF), on atomic clusters from simplified nanoindentation tests.

Main conclusions of this work are summarized below:

1. Our MLIP accurately reproduced finite-temperature *ab initio* energies of AlB_2_-type TiB_2−*x*_ structures with point or planar defect types and contents not directly trained on (RMSE_max_ ≈ 6 meV at^−1^). Further, it showed good agreement with *ab initio* and—where applicable—experimental lattice parameters (±0.02%) and second-order elastic constants (±10%), as well as reliable extrapolation grades during mechanical tests at both AIMD length scales (uniaxial tensile or shear simulations under fully periodic boundary conditions) and beyond (nanoindentation).

2. Room-temperature tensile tests for supercells with ≈140 000 atoms, uni-axially loaded along [0001], [1̄21̄0], or [101̄0] direction, ascribed the highest theoretical strength (54.5 GPa) to the stoichiometric TiB_2_. In B-sub-stoichiometric structures, the strength gradually reduced by 57%, 53% and 28% in case of vacancies, single-planar defects and double-planar defects, respectively, as B content decreased from the ideal TiB_2_ to TiB_1.5_. Notably, the most significant reduction occurred in the direction normal to the planar defect, associated with brittle cleavage perpendicular to Ti-rich (101̄0) planes. For the shear test along the (0001)[101̄0] slip system, the perfect TiB_2_ model exhibited the highest shear strength but only an intermediate slip strain compared with all other tested models.

3. Room-temperature nanoindentation tests—considering commonly reported [0001]-oriented samples—showed a significant hardness and modulus decrease for TiB_2−*x*_ with disordered B vacancies: by 48% and 37%, respectively, for TiB_1.5_, as compared with the ideal TiB_2_. Contrarily, the *H* decrease for TiB_2−*x*_ with Ti-rich planar defects was way less dramatic, about 8%, for TiB_1.5_ with single-planar defects. TiB_1.7_ with double-planar defects exhibited *H* = 66 GPa even slightly above *H* = 65 GPa of the ideal TiB_2_. The predicted *H* values were generally 10%–20% above experimental data, as our models only account for one defect type. Further, structural analysis suggested that planar defects serve as barriers impeding the dislocation glide or slip beneath the indentation region, thereby contributing to the increased *H* or preventing a rapid *H* decrease.

Our study indicated that MLIPs transferable to complex stress states near indenter tips require on-the-fly training, additionally to training on bulk and surface structures from *ab initio* MD. The here-proposed training strategy can be straightforwardly applied to other AlB_2_-type diborides and is likely suitable also for transition metal carbides and nitrides (typically crystallizing with different, cubic, crystal symmetry). Further, the wide spread of *H* data and the fact that largely B-substoichiometric compositions—though less energetically favorable compared with the fully occupied lattice—can even exceed *H* of the ideal TiB_2_ was explained by different mechanical response of TiB_2−*x*_ with B vacancies and Ti-rich planar defects. We hypothesize that similar *H* trends and non-stoichiometry driven deformations mechanisms can be found in diborides energetically favoring the AlB_2_ structure, particularly ZrB_2_ and HfB_2_ isoelectronic to TiB_2_.

All in all, the unique capability of atomistic simulations to evaluate effects of individual defect types, for desired chemical compositions, under well defined and comparable loading conditions, was demonstrated. Challenges for follow-up research include modeling more complex (combinations of) defects, such as grain boundaries or amorphous B-rich tissue phase frequently reported in diborides. Following our MLIP training and MD simulation routine, a fundamentally interesting question is whether TM-rich planar defects have similar effects in other TMB_2_s and can be used in rational structural design.

## Author contributions

CD: conceptualization, methodology, investigation, data curation, visualization, writing – original draft. SL: investigation, validation, writing – review & editing. NK: conceptualization, methodology, writing – review & editing, supervision. PHM: resources, writing – review & editing, supervision.

## Conflicts of interest

The authors declare that they have no known competing financial interests or personal relationships that could have appeared to influence the work reported in this paper.

## Supplementary Material

NR-018-D6NR00466K-s001

## Data Availability

The supporting data has been provided as part of the supplementary information (SI). Supplementary information: Table S1, Fig. S1, S2 and S3. See DOI: https://doi.org/10.1039/d6nr00466k. The relevant MLIP and lattice structures are stored in the TU Wien Research Data repository. See DOI: https://doi.org/10.48436/3mat6-bp059.
