# Supplementary material for: Towards nano-mechanical simulations of ceramics containing realistic defects via machine-learning potentials: the example of TiB2
Source: Nanoscale. 2026 Jun 10;18(27):14397–410. doi: 10.1039/d6nr00466k (PMC13250926; doi:10.1039/d6nr00466k)
Supplement: NR-018-D6NR00466K-s001 [file NR-018-D6NR00466K-s001.pdf]

Supplementary Information (SI) for Nanoscale.
This journal is © The Royal Society of Chemistry 2026

Supplementary Materials

Towards nano-mechanical simulations of ceramics containing realistic defects via machine-learning potentials: the example of TiB_2

Chunhui Du[1*], Shuyao Lin[1,2], Nikola Koutná[1,2] and Paul Heinz Mayrhofer[1]

[1]Institute of Material Science and Technology, TU Wien, Vienna, Austria

[2]Department of Physics, Chemistry, and Biology (IFM), Linköping University, Sweden

* chunhui.du@tuwien.ac.at

S1. Theoretical mechanical properties of models

Models	ν	σ_{max} (GPa)			ϵ_{max} (%)			τ_{max} (GPa)	γ_{max} (%)
		[0001]	$[10\bar{1}0]$	$[\bar{1}2\bar{1}0]$	[0001]	$[10\bar{1}0]$	$[\bar{1}2\bar{1}0]$		
$TiB_{1.5}$-SP	0.183	24.30	16.96	31.59	8.6	5.4	8.6	16.9	30.9
$TiB_{1.5}$-DP	0.161	37.08	14.63	38.13	16.0	6.4	10.8	25.4	38.0
$TiB_{1.5}$-Vac	0.206	22.52	25.14	25.52	12.9	10.4	10.3	13.0	11.9
$TiB_{1.6}$-SP	0.142	32.72	20.02	35.09	11.4	5.4	8.9	27.3	20.8
$TiB_{1.6}$-DP	0.140	39.13	15.03	40.78	15.8	5.3	11.2	25.4	35.8
$TiB_{1.6}$-Vac	0.171	28.90	30.12	31.14	13.9	11.2	11.0	17.4	12.2
$TiB_{1.7}$-SP	0.146	46.70	22.20	49.29	23.2	5.7	15.9	33.3	16.5
$TiB_{1.7}$-DP	0.151	37.71	14.77	42.09	14.2	5.0	11.2	24.4	33.3
$TiB_{1.7}$-Vac	0.174	32.30	32.58	35.16	14.9	11.5	11.9	21.5	13.2
$TiB_{1.8}$-SP	0.131	48.21	25.49	52.17	23.7	6.9	17.1	33.2	15.3
$TiB_{1.8}$-DP	0.126	42.80	15.32	44.28	17.4	4.1	12.2	26.0	35.1
$TiB_{1.8}$-Vac	0.145	38.69	36.98	39.36	16.7	12.6	12.5	25.0	13.1
TiB_2	0.138	51.94	46.22	54.49	24.6	14.2	16.9	48.2	21.8

Tab. S1: Theoretical Poisson's ratio (ν), maximum uniaxial fracture strength (σ_{max}), fracture strain (ϵ_{max}), shear strength (τ_{max}) and shear strain (γ_{max}) from nano-scale uniaxial tensile tests, computed using the ideal stoichiometric model and off-stoichiometric models.

S2. Stress-strain curve of defective models

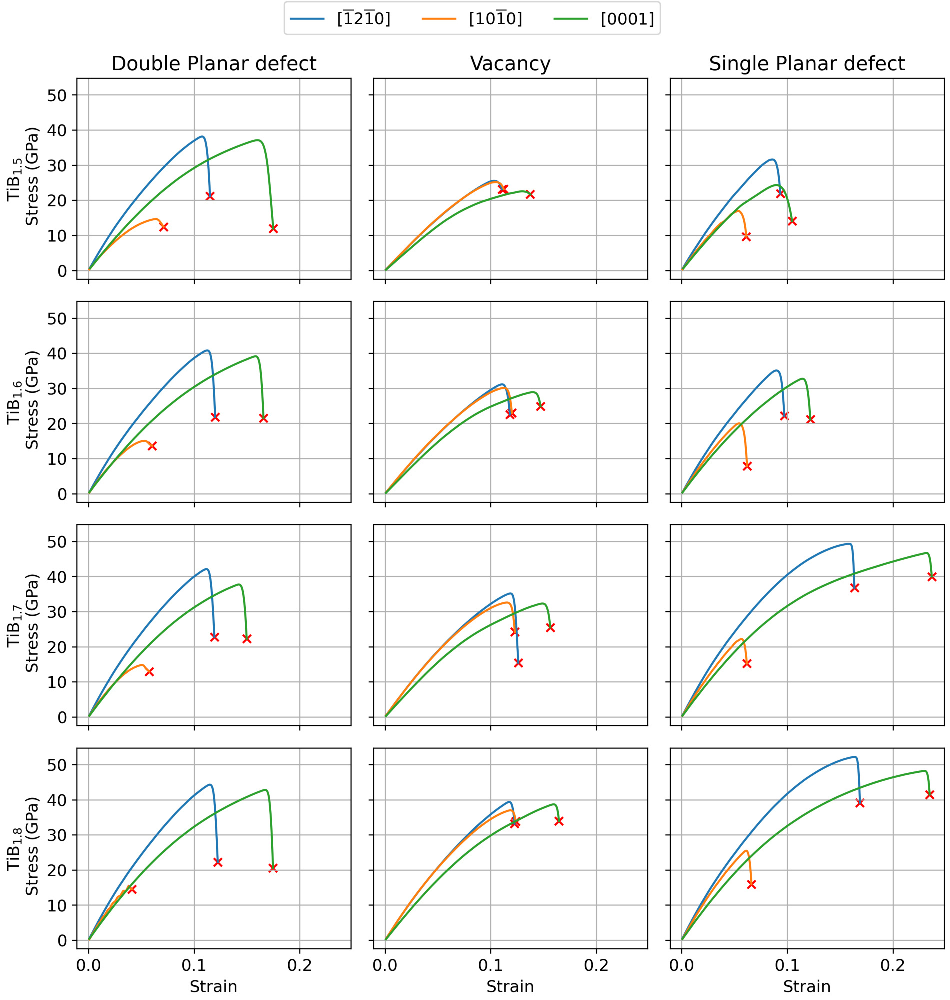

Fig. S1: Comparison of the stress–strain curves for defective TiB_{2-x} models with various defect types. The data are obtained from large-scale molecular dynamics simulations of uniaxial tensile tests performed along three different crystallographic directions: [$\bar{1}2\bar{1}0$], [$10\bar{1}0$] and [0001], represented by lines of different colors.

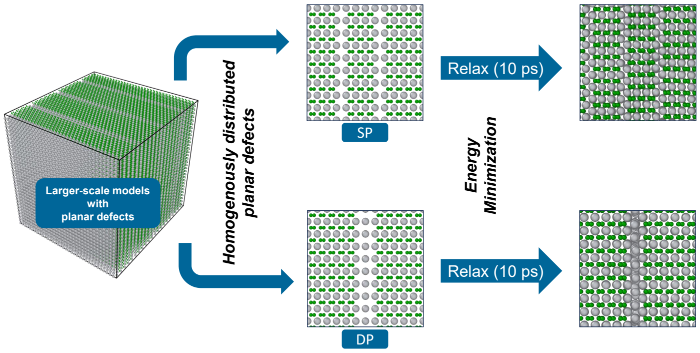

Fig. S2: Large-scale simulation models with planar defects used in LAMMPS: Comparison before and after energy minimization (relaxation). SP: Single planar defect; DP: Double planar defect

S3. PDOS (Partial Density of States) calculation results of defective models

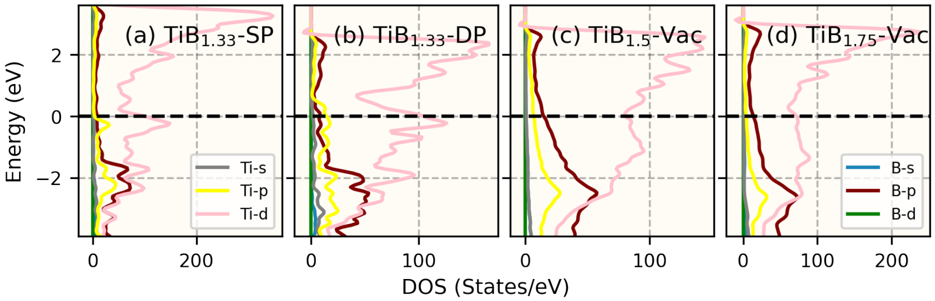

Fig. S3: Electronic density of states (DOS) for TiB_x systems with varying compositions and defect configurations: (a) $TiB_{1.33}$-SP, (b) $TiB_{1.33}$-DP, (c) $TiB_{1.5}$-Vac, and (d) $TiB_{1.75}$-Vac. The total and orbital-projected DOS (s, p, and d states) are shown for Ti (gray, yellow, pink) and B (blue, red, green) atoms, as indicated in the legends. The energy scale is referenced to the Fermi level, set to 0 eV (black dashed line). Positive and negative energies correspond to unoccupied and occupied states, respectively. SP: Single planar defect; DP: Double planar defect; Vac: random distributed boron vacancies
